# Supplementary material for: Addressing imbalanced data classification with Cluster-Based Reduced Noise SMOTE
Source: PLoS One. 2025 Feb 10;20(2):e0317396. doi: 10.1371/journal.pone.0317396 (PMC11809912; doi:10.1371/journal.pone.0317396)
Supplement: Attachment — Submitted filename: Response to Reviewers.docx [file pone.0317396.s002.docx]

Manuscript number: ***PONE-D-24-38797***

Title: ***Addressing Imbalanced Data Classification with Cluster-Based Reduced Noise SMOTE***

*Authors*: *Javad Hemmatian, Rassoul Hajizadeh and Fakhroddin Nazari*

*Dear Reviewers,*

The authors would like to express their sincere appreciation to the reviewers for the time and efforts that have gone into their detailed reviews. We have carefully revised the manuscript to address all comments of the reviewers directly. The corresponding changes and refinements made in the revised manuscript are summarized in our responses below, and can be easily tracked in the revised manuscript.

Sincerely,

*Rassoul Hajizadeh*

***Reviewers' comments:***

***Reviewer #1:***

***Comment* 1:** The author mentions which dataset results have better accuracy in the abstract.

***Answer 1:***

We appreciate the reviewer’s feedback regarding the abstract. In revised manuscript, we have revised the abstract to specify which datasets demonstrated better accuracy results. Specifically, we highlighted the improved performance of the CRN-SMOTE method on the QSAR and Maternal Health Risk datasets, along with a general statement about its effectiveness across all evaluated datasets.

***Comment* 2:** The literature review has two subheadings current research and research gap.

***Answer 2:***

The literature review presented in the Introduction section has been thoroughly justified, aiming to provide a complete and comprehensive overview of existing research. The changes are highlighted in revised manuscript.

***Comment* 3:** The author needs to read the following manuscript and add some applications of Machine learning in different fields in the literature review ”Enhancing software defect prediction: a framework with improved feature selection and ensemble machine learning” “Impact of 3G and 4G technology performance on customer satisfaction in the telecommunication industry” Software defect prediction using an intelligent ensemble-based model.

***Answer 3:***

We have reviewed and studied the above manuscripts and with the help of the mentioned articles we have improved our manuscript by incorporating different machine learning programs. In references 6, 7, and 8, we incorporate new applications of machine learning discussed in the aforementioned articles into our manuscript.

***Comment* 4:** Future work must be a section

***Answer 4:***

We improved the future work section and designated it as a separate section, which we placed in section 5***.***

***Comment* 5:** The author needs to add a table before the conclusion in which results have been compared with some other studies

***Answer 5:***

In the Simulation Results section, the performance of the proposed CRN-SMOTE (i.e., 1CRN-SMOTE) is compared with two state-of-the-art SMOTE-based methods, SMOTE-Tomek Link and SMOTE-ENN, as well as the RN-SMOTE approach in the revised manuscript.

“The results are provided for $K_{SMOTE}$=5 and the RF classifier across all four mentioned datasets (see Tables 7 and 8). The findings demonstrate that the proposed CRN-SMOTE method outperforms other state-of-the-art methods in most cases. On the Blood dataset, SMOTE-ENN outperforms the proposed CRN-SMOTE method, while on all three other datasets, the proposed 1CRN-SMOTE demonstrates the best performance.”

**Table 7.** A comparison of the RN-SMOTE, SMOTE-Tomek Link, SMOTE-ENN, and the proposed 1CRN-SMOTE methods on the ILPD and QSAR datasets is presented, based on various classification metrics using the Random Forest classifier.

| ***Dataset*** | | | | | | | | | |  |
| --- | --- | --- | --- | --- | --- | --- | --- | --- | --- | --- |
| ***QSAR*** | | | | | ***ILPD*** | | | | |  |
| **Recall** | **Pre** | **F1** | **MCC** | **kappa** | **Recall** | **Pre** | **F1** | **MCC** | **kappa** | *method* |
| **0.892** | **0.892** | **0.891** | **0.757** | **0.756** | **0.723** | **0.740** | **0.727** | **0.358** | **0.352** | Proposed method  (1CRN-SMOTE) |
| 0.852 | 0.856 | 0.853 | 0.677 | 0.675 | 0.617 | 0.739 | 0.632 | 0.309 | 0.264 | SMOTE-ENN [] |
| 0.877 | 0.876 | 0.876 | 0.723 | 0.722 | 0.687 | 0.707 | 0.687 | 0.280 | 0.274 | SMOTE-TOMEK LINK [] |
| 0.877 | 0.876 | 0.876 | 0.722 | 0.719 | 0.711 | 0.727 | 0.715 | 0.327 | 0.322 | RN-SMOTE[] |

**Table 8.** A comparison of the RN-SMOTE, SMOTE-Tomek Link, SMOTE-ENN, and the proposed 1CRN-SMOTE methods on the Blood and Health-risk datasets is presented, based on various classification metrics using the Random Forest classifier

| ***Dataset*** | | | | | | | | | |  |
| --- | --- | --- | --- | --- | --- | --- | --- | --- | --- | --- |
| ***Health-risk*** | | | | | ***Blood*** | | | | |  |
| **Recall** | **Pre** | **F1** | **MCC** | **kappa** | **Recall** | **Pre** | **F1** | **MCC** | **kappa** | *method* |
| **0.862** | **0.868** | **0.862** | **0.795** | **0.792** | 0.698 | 0.711 | 0.703 | 0.201 | 0.200 | Proposed method  (1CRN-SMOTE) |
| 0.7297 | 0.731 | 0.723 | 0.594 | 0.587 | **0.717** | **0.762** | **0.730** | **0.330** | **0.317** | SMOTE-ENN [] |
| 0.850 | 0.855 | 0.862 | 0.775 | 0.772 | 0.700 | 0.709 | 0.702 | 0.197 | 0.196 | SMOTE-TOMEK LINK [] |
| 0.849 | 0.854 | 0.848 | 0.774 | 0.771 | 0.707 | 0.705 | 0.703 | 0.184 | 0.182 | RN-SMOTE[] |

The tables and paragraph above have been added to the revised manuscript.

***Comment* 6:** There must be a discussion section at the end

***Answer 6:***

In line with the respected referee's suggestion, we have positioned the discussion section at the end of Section 4 (i.e., section 4.4) to offer a more comprehensive analysis of our findings.

***Comment* 7:** The author needs to add tables and figures in the Manuscript rather than down the manuscript so that there will be a better look and easier for the reader

***Answer 7:***

we completely agree with your suggestion. However, due to the limitations set by the editor during the initial submission stage, we were instructed to remove the figures from the main manuscript. Below is the editor's quote from the time of submission:

"Do not include figures in the main manuscript file. Each figure must be prepared and submitted as an individual file.

Cite figures in ascending numeric order at first appearance in the manuscript file."

***Comment* 8:** What are the issues of the imbalanced dataset and how was it resolved

***Answer 8:***

Imbalanced datasets can lead to several issues, including:

***a) Bias toward majority class****:* Models may become biased toward the majority class, resulting in poor performance on the minority class.

***b) Misleading Accuracy***: High overall accuracy can be misleading, as it may reflect the model's ability to predict the majority class rather than its effectiveness in identifying the minority class.

***c) Poor Generalization***: The model may struggle to generalize to new, unseen data, particularly for the minority class.

***d) Increased False Negatives***: There is a higher likelihood of misclassifying minority class instances, leading to increased false negatives.

Generally, there are three primary approaches to address this issue, as illustrated in Figure 1: (1) data-level techniques, (2) algorithm-level approaches, and (3) hybrid methods that combine both. The details of these approaches are explained in the Introduction section of the revised manuscript.

**Figure 1.** Taxonomy of approaches to addressing imbalanced data issue.

The issues mentioned have been incorporated into the revised manuscript.

***Comment* 9:** Write the advantages and disadvantages of the proposed method.

***Answer 9:***

The proposed method, Cluster-Based Reduced Noise SMOTE (CRN-SMOTE), aims to improve the performance of classification algorithms when dealing with imbalanced datasets. It combines the Synthetic Minority Oversampling Technique (SMOTE) with a novel cluster-based noise reduction technique. Here are the key advantages and disadvantages of this method:

***Advantages***:

***a) Improved Classification Performance***: CRN-SMOTE consistently outperforms existing methods, such as Reduced Noise SMOTE (RN-SMOTE), SMOTE-ENN, and SMOTE Tomek Link across various metrics like Cohen's kappa, Matthew’s correlation coefficient, F1-score, precision, and recall. This indicates a more effective handling of imbalanced data, leading to better predictive accuracy for minority classes.

***b)* *Cluster control in noise Reduction step***: The cluster-based approach effectively reduces noise in the data by ensuring that samples from each category form one or two clusters. This is crucial because it prevents the misclassification of minority class samples that could occur due to noise in the dataset.

***c)* *Focus on Classification Applications***: Unlike many traditional data-level methods that merely aim to balance datasets without considering classification implications, CRN-SMOTE is specifically designed with classification performance in mind. This results in a more targeted approach to addressing imbalanced data issues.

***Disadvantages***:

***a)* *Complexity of Implementation***: While the method's focus on clustering can enhance performance, it may also introduce complexity in implementation. Understanding and correctly applying clustering techniques may require additional expertise and computational resources.

***b)* *Dependence on Clustering Quality***: The effectiveness of CRN-SMOTE is contingent upon the quality of the clustering process. If the clustering parameters are not optimally set or if the underlying data structure is complex, it may lead to poor performance or misclassification.

***Comment* 10:** What are the disadvantages of the Synthetic Minority Oversampling Technique

***Answer 10:***

*a)* One challenge posed by SMOTE is the potential for overfitting, particularly when the minority class is small. In such cases, the model may learn noise rather than generalizable patterns. This issue is often addressed by applying denoising techniques after the SMOTE step.

*b)* Increased Complexity: SMOTE increases the size of the dataset, which can lead to longer training times and greater computational resource requirements. This can be a disadvantage in scenarios where quick model training is needed*.*

*c)* Noise Amplification: If the minority class contains noisy or mislabeled instances, SMOTE can amplify this noise by generating synthetic samples based on these problematic instances, further complicating the classification task.

*d)* Class Proximity: SMOTE does not consider the distribution of the majority class. If the majority class is very close to the minority class in feature space, the synthetic samples may lie too close to the majority class, increasing the risk of misclassification.

Briefly, the disadvantages of the SMOTE are illustrated in Figure 2.

**Figure 2.** The disadvantages of the SMOTE

***Comment* 11:** The conclusion must be concise as abstract

***Answer 11:***

The conclusion has been revised.

***Reviewer #2:***

***Comment* 1:** The abstract needs more improvement, the authors should mention the results and improvement than existing methods.

***Answer 1:***

The abstract has been revised and improved.

***Comment* 2:** The problem statement and motivation of the paper needs to be clearly mention to provide a clear background to the readers. 2. The authors should provide the main contributions in points at the end of introduction section.

***Answer 2:***

The Contribution and Motivation section has been modified and added to the end of the Introduction section. Additionally, we have clearly outlined the main contributions of the proposed method in bullet points at the conclusion of the Introduction in the revised manuscript.

***Comment* 3:** I appreciate the valuable use of machine learning in the model. For the readers concerns, I suggest adding/citing the recent computational models related machine learning and oversampling such as AIPs-DeepEnC-GA, StackedEnC-AOP, DeepAVPTPPred, iAFPs-Mv-BiTCN, AIPs-SnTCN and Deepstacked-AVPs in order to provide useful information to the readers.

***Answer 3:***

Thank you for your valuable suggestions. We have incorporated most of them into the revised manuscript.

***Comment* 4:** How the authors handle the over fitting and generalization of the proposed model.

***Answer 4:***

Overfitting in imbalanced data classification refers to a situation where a model learns to perform exceptionally well on the training data, including the majority class, but fails to generalize to unseen data, particularly the minority class. In imbalanced datasets, where one class (usually the majority) significantly outnumbers the other (minority), the model may focus excessively on the majority class.

The proposed CRN-SMOTE addresses this issue at the data level by oversampling the minority categories. The method consists of three steps: 1. Oversampling using the SMOTE technique, 2. Cluster-based noise reduction, and 3. Final oversampling with SMOTE. These steps create a balanced dataset across all categories. By equalizing the sample sizes of each category, the risk of overfitting can be mitigated.

***Comment* 5:** The authors should clearly mention the future directions and real-life applications of the proposed study.

***Answer 5:***

*Future work* Section has been added to the revised manuscript. Also, some applications of the proposed study have been presented in the Introduction section of the revised manuscript.

***Comment* 6:** What are the limitations of the proposed model.

***Answer 6:***

The following issues can be regarded as limitations of the proposed method:

*a) Complexity of Implementation:* While the method's focus on clustering can enhance performance, it may also introduce complexity in implementation. Understanding and correctly applying clustering techniques may require additional expertise and computational resources.

*b) Dependence on Clustering Quality:* The effectiveness of CRN-SMOTE is contingent upon the quality of the clustering process. If the clustering parameters are not optimally set or if the underlying data structure is complex, it may lead to poor performance or misclassification.
